# Assessment of epigenetic alterations in early colorectal lesions containing *BRAF* mutations

**DOI:** 10.18632/oncotarget.9044

**Published:** 2016-04-27

**Authors:** Takeshi Sawada, Eiichiro Yamamoto, Hiro-o Yamano, Masanori Nojima, Taku Harada, Reo Maruyama, Masami Ashida, Hironori Aoki, Hiro-o Matsushita, Kenjiro Yoshikawa, Eiji Harada, Yoshihito Tanaka, Shigenori Wakita, Takeshi Niinuma, Masahiro Kai, Makoto Eizuka, Tamotsu Sugai, Hiromu Suzuki

**Affiliations:** ^1^ Department of Molecular Biology, Sapporo Medical University School of Medicine, Sapporo, Japan; ^2^ Department of Advanced Research in Community Medicine, Kanazawa University Graduate School of Medical Sciences, Kanazawa, Japan; ^3^ Department of Gastroenterology, Rheumatology and Clinical Immunology, Sapporo Medical University School of Medicine, Sapporo, Japan; ^4^ Department of Gastroenterology, Akita Red Cross Hospital, Akita, Japan; ^5^ Center for Translational Research, The Institute of Medical Science, The University of Tokyo, Tokyo, Japan; ^6^ Division of Cardiovascular Medicine, Department of Internal Medicine, Kanazawa University Graduate School of Medicine, Kanazawa, Japan; ^7^ Department of Molecular Diagnostic Pathology, Iwate Medical University School of Medicine, Morioka, Japan

**Keywords:** colorectal cancer, BRAF, serrated lesion, methylation, CpG island methylator phenotype

## Abstract

To clarify the molecular and clinicopathological characteristics of colorectal serrated lesions, we assessed the DNA methylation of cancer-associated genes in a cohort of *BRAF*-mutant precancerous lesions from 94 individuals. We then compared those results with the lesions' clinicopathological features, especially colorectal subsites. The lesions included hyperplastic polyps (n = 16), traditional serrated adenomas (TSAs) (n = 15), TSAs with sessile serrated adenomas (SSAs) (n = 6), SSAs (n = 49) and SSAs with dysplasia (n = 16). The prevalence of lesions exhibiting the CpG island methylator phenotype (CIMP) was lower in the sigmoid colon and rectum than in other bowel subsites, including the cecum, ascending, transverse and descending colon. In addition, several cancer-associated genes showed higher methylation levels within lesions in the proximal to sigmoid colon than in the sigmoid colon and rectum. These results indicate that the methylation status of lesions with *BRAF* mutation is strongly associated with their location, histological findings and neoplastic pathways. By contrast, no difference in aberrant DNA methylation was observed in normal-appearing background colonic mucosa along the bowel subsites, which may indicate the absence of an epigenetic field defect.

## INTRODUCTION

Recent studies have shown that colorectal cancers (CRCs) are heterogeneous diseases that derive from distinct molecular pathways [1, 2]. A large proportion (80-85%) of sporadic CRCs develop through accumulation of multiple genetic and epigenetic alterations, including mutation of oncogenes and tumor suppressor genes [3], chromosomal instability (CIN) and global DNA hypomethylation. The remaining 15-20% of sporadic CRCs exhibit microsatellite instability (MSI) and concurrent hypermethylation in multiple loci, which is referred to as the CpG island methylator phenotype (CIMP) [4] and is tightly associated with *BRAF* mutation [5, 6].

Similarly, molecular and clinicopathological analysis of colorectal premalignant lesions, including conventional adenomas and serrated lesions, has provided insight into the development of CRC and its implications for prevention and treatment [7]. Since establishment of the pathological classification of serrated colorectal lesions as hyperplastic polyps (HPs), traditional serrated adenomas (TSAs) or sessile serrated adenomas (SSAs) [8–11], there have been several investigations of the molecular alterations within those lesions. Those studies provide evidence that SSAs are associated with *BRAF* mutation and CIMP, and that they are precursors of MSI-positive CRCs, which preferentially locate in the proximal colon (serrated-neoplasia pathway) [6, 12–14]. TSAs are also considered to be premalignant lesions and reportedly exhibit *BRAF* or *KRAS* mutations and aberrant DNA methylation [11, 15–20], though their biological and clinical characteristics are not yet fully understood. One aim of the present study was to clarify the involvement of epigenetic alterations in serrated lesions.

The two-side colon theory was recently challenged by the observation that the frequencies of *BRAF* mutation, CIMP and *MLH1* methylation in CRCs gradually increase along the colon, from the rectum to the ascending colon [21]. However, there have been few reported studies on the association between colorectal subsites and the clinicopathological and molecular characteristics in precancerous lesions [22, 23]. In the present study, we also assessed the association between DNA methylation status and clinicopathological features, including tumor locations in early colorectal lesions with *BRAF* mutation.

Epigenetic fields for cancerization (also known as “epigenetic field defects”) have been reported in several cancers, including CRC [24]. Since the first report of estrogen receptor gene methylation in normal colorectal mucosa [25], DNA methylation of a number of genes in normal-appearing colorectal mucosa from patients with CRC [26–29] or a precursor lesion [29,30] have been evaluated. However, the methylation status of the normal mucosa adjacent to serrated lesions has not been studied in detail. We therefore addressed this issue in *BRAF*-mutant precursor lesions.

## RESULTS

### CIMP status and locations of *BRAF*-mutant lesions

**Figure 1:**
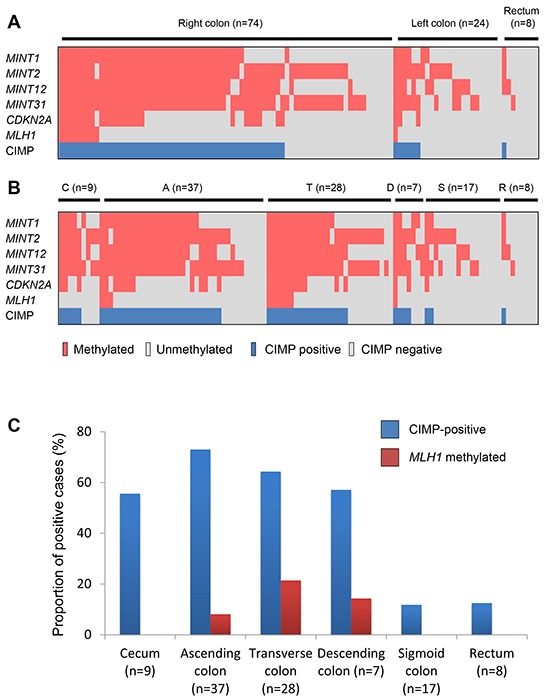
Methylation profiles in *BRAF*-mutant lesions **A.** Methylation of CIMP markers and CIMP status in lesions in the right colon (from cecum to transverse colon) and the left colon (from descending colon to sigmoid colon). **B.** Methylation status in *BRAF*-mutant lesions in the cecum (C) and ascending (A), transverse (T), descending (D) and sigmoid (S) colon and rectum (R). **C.** Frequencies of CIMP and *MLH1* methylation in *BRAF*-mutant lesions in the indicated bowel subsites.

The clinicopathological and molecular characteristics of the early colorectal lesions with *BRAF* mutation analyzed in this study are summarized in Tables 1 and 2. The majority (74/106, 69.8%) of these lesions were located in the right colon (from the cecum to the transverse colon), while 24 lesions (22.7%) were found in the left colon (descending and sigmoid colon), and 8 (7.5%) were in the rectum (Table 1). SSAs were predominantly observed in the proximal colon, while TSAs were more prevalent in the sigmoid colon and rectum (Table 2). The CIMP statuses of the lesions in the respective bowel subsites are summarized in Figure 1. Notably, CIMP-positive lesions were located predominantly in the proximal bowel subsites, from the cecum to the descending colon, and were significantly less frequent in the sigmoid colon and rectum (Figure 1C). Logistic regression analysis confirmed that the correlation between tumor location and CIMP status was most significant when the bowel was subdivided using the sigmoid-descending colon junction as a boundary (Table 3).

**Table 1:** Clinicopathological features of the *BRAF*-mutant lesions in this study

Patients (n=94)	
**Age (years, mean ± SD)**	62.9 ± 11.3
**Gender**	
Female	36 (38.3%)
Male	58 (61.7%)
**Lesions (n=106)**	
**Location**	
Right colon	74 (69.8%)
Left colon	24 (22.7%)
Rectum	8 (7.5%)
**Bowel subsites**	
Cecum	9 (8.5%)
Ascending colon	37 (34.9%)
Transverse colon	28 (26.4%)
Descending colon	7 (6.6%)
Sigmoid colon	17 (16.0%)
Rectum	8 (7.6%)
**Morphology**	
Flat	62 (58.5%)
Protruded	41 (38.7%)
Flat+protruded	3 (2.8%)
**Histology**	
HP/IM	16 (15.1%)
TSA	15 (14.2%)
TSA+SSA	6 (5.7%)
SSA	49 (46.2%)
SSA+CD	9 (8.5%)
SSA+HGD	7 (6.6%)
Conventional adenoma	3 (2.8%)
HGD	1 (0.9%)

HP, hyperplastic polyp; IM, intermediate; TSA, traditional serrated adenoma; SSA, sessile serrated adenoma; CD, cytological dysplasia; HGD, high grade dysplasia

**Table 2:** Clinicopathological and molecular characteristics of *BRAF*-mutant lesions

	Total number	Proximal colon			Distal colon		Rectum
		Cecum	Ascending colon	Transverse colon	Descending colon	Sigmoid colon	
**Patients**	94	9	31	25	7	14	8
**Age (years, mean ± SD)**	62.9 ± 11.3	63.9 ± 8.3	65.8 ± 10.7	65.1 ± 12.9	60.3 ± 6.9	56.1 ± 12.4	65.0 ± 8.7
**Sex**							
Female	36 (38.3%)	5	13	15	1	2	0
Male	58 (61.7%)	4	18	10	6	12	8
**Lesions**	106	9	37	28	7	17	8
**Morphology**							
Flat	62 (58.5%)	8	28	18	2	4	2
Protruded	41 (38.7%)	1	8	9	5	13	5
Flat+protruded	3 (2.8%)		1	1			1
**Size (mm, mean ± SD)**	10.2 ± 5.6	10.3 ± 5.8	11.7 ± 5.9	11.1 ± 5.8	7.7 ± 1.8	8.2 ± 4.9	6.5 ± 2.1
**Histology**							
HP/IM	16 (15.1%)		3	3	1	6	3
TSA	15 (14.2%)		1	1	2	8	3
TSA+SSA	6 (5.7%)		3	3			
SSA	49 (46.2%)	9	20	14	3	2	1
SSA+CD	9 (8.5%)		5	4			
SSA+HGD	7 (6.6%)		3	3	1		
Conventional adenoma	3 (2.8%)		1			1	1
HGD	1 (0.9%)		1				
**CIMP status**							
Positive	57 (53.8%)	5	27	18	4	2	1
Negative	49 (46.2%)	4	10	10	3	15	7
***MLH1* methylation**							
Positive	10 (12.9%)	0	3	6	1	0	0
Negative	96 (87.1%)	9	34	22	6	17	8
**Adjacent normal colon**	83	8	28	22	7	12	6

HP, hyperplastic polyp; IM, intermediate; TSA, traditional serrated adenoma; SSA, sessile serrated adenoma; CD, cytological dysplasia; HGD, high grade dysplasia

**Table 3:** Correlations between tumor location and CIMP status in *BRAF*-mutant lesions

Demarcation	OR (95% CI)	*P*
C-A	1.39 (0.36-5.46)	0.447
A-T	2.9 (1.37-6.13)	0.004
T-D	7.14 (2.81-18.18)	0.000011
D-S	13.89 (3.91-50.00)	0.000001
S-R	9.52 (1.16-76.92)	0.018

C, cecum; A, ascending colon; T, transverse colon; D, descending colon; S, sigmoid colon; R, rectum

**Figure 2:**
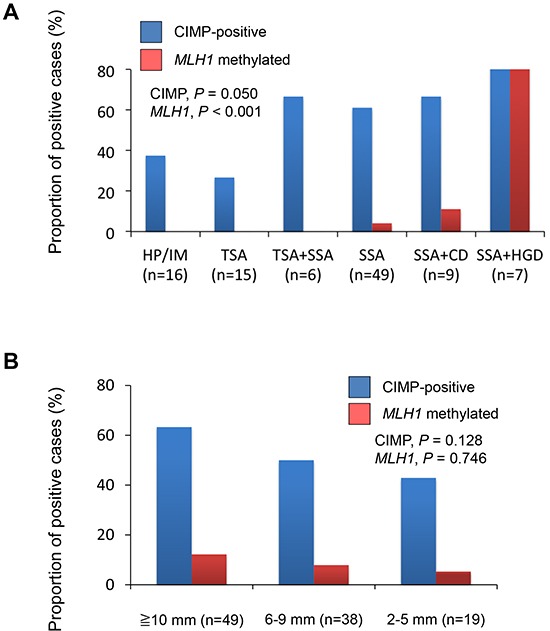
**A.** Frequencies of CIMP and *MLH1* methylation in lesions with the indicated histological findings: HP, hyperplastic polyp; IM, intermediate; TSA, traditional serrated adenoma; SSA, sessile serrated adenoma; CD, cytological dysplasia; HGD, high-grade dysplasia. **B.** Frequencies of CIMP and *MLH1* methylation in *BRAF*-mutant lesions with indicated diameters.

We next assessed the relationship between CIMP status and the clinicopathological findings (Table 2). CIMP was detected more frequently in lesions containing SSA components (SSAs, 61.2%; SSAs with cytological dysplasia, 66.7%; SSA with high-grade dysplasia, 85.7%; TSA with SSAs, 66.7%) than in TSAs (26.7%) or HPs/intermediate (IM), which is serrated lesions that did not satisfy the criteria for SSA or TSA (37.5%) (Figure 2A). Consistent with an earlier report [23], we found a tendency for CIMP to be more prevalent among larger tumors, though this was not statistically significant (Figure 2B).

Methylation of *MLH1* is a hallmark of MSI in sporadic CRCs. We assessed MSI status in 24 lesions and found that the methylation status of *MLH1* was consistent with the MSI status in 23 of 24 lesions (7 of 24 were MSI-positive, while 6 were *MLH1* methylation-positive; P < 0.001). We observed *MLH1* methylation in lesions within the ascending, transverse and descending colon, but its frequency was much lower than that of CIMP (Figure 1C). Moreover, *MLH1* methylation was observed much more frequently in SSA+HGDs (85.7%) than in other types of lesions (SSA+CDs, 6.7%; SSAs, 4.1%; TSA+SSAs, 0%; TSAs, 0%; HP/IMs. 0%) (Figure 2A). These results are consistent with the earlier observations that SSAs are precursor lesions for CIMP-positive and MSI-positive CRCs, and that *MLH1* methylation is not an early event during colorectal tumorigenesis [6, 31, 32].

### Methylation of tumor-related genes in *BRAF*-mutant lesions

**Figure 3:**
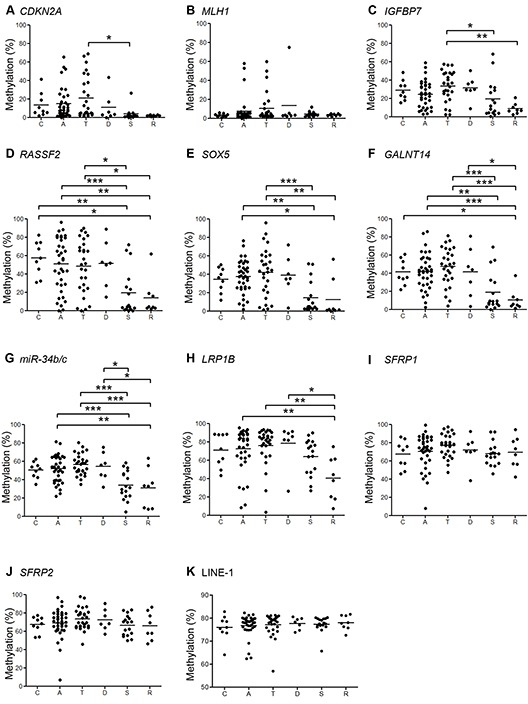
Summaries of the bisulfite pyrosequencing results from *BRAF*-mutant lesions Shown are the levels of methylation of indicated genes **A–H.** and LINE-1 **I.** in lesions in the cecum (C) and ascending (A), transverse (T), descending (D) and sigmoid (S) colon and rectum (R). *P < 0.05, **P < 0.01, ***P < 0.001.

We next assessed methylation of the promoter CpG islands of genes known to be frequently methylated in CRC [14]. We found that the methylation levels of *CDKN2A*, *IGFBP7*, *RASSF2*, *SOX5*, *GALNT14*, *miR-34b/c* and *LRP1B* were significantly higher in lesions located at bowel subsites from the cecum to the descending colon than in lesions in the sigmoid colon and rectum, which was consistent with the CIMP status (Figure 3, Supplementary Figure S1). By contrast, levels of *SFRP1* and *SFRP2* methylation were consistently elevated in lesions throughout the entire bowel, indicating that those genes are methylated, irrespective of tumor location or the molecular subtype of the tumor.

To evaluate global DNA hypomethylation within the lesions, we analyzed levels of LINE-1 methylation, which did not correlate with bowel location (Figure 3K, Supplementary Figure S1K). Nor did levels of LINE-1 methylation correlate significantly with CIMP status or *MLH1* methylation (data not shown).

### Methylation analysis of adjacent normal-appearing mucosae

**Figure 4:**
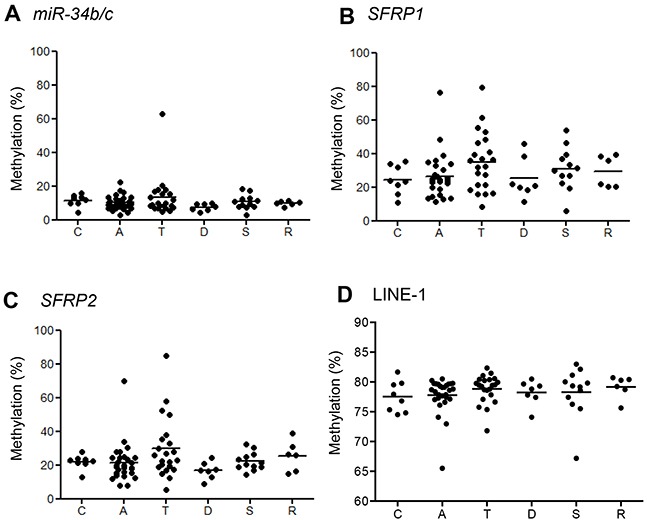
Summaries of bisulfite pyrosequencing results in normal-appearing mucosa adjacent to *BRAF*-mutant lesions Shown are levels of methylation of the indicated genes **A–C.** and LINE-1 **D.** in the cecum (C) and ascending (A), transverse (T), descending (D) and sigmoid (S) colon and rectum (R).

To determine whether an epigenetic field defect is involved in the development of *BRAF*-mutant lesions, we collected matched samples of the lesion and normal-appearing colonic mucosa adjacent to the lesions from 83 individuals and then assessed the methylation status of three selected genes (*miR-34b/c*, *SFRP1* and *SFRP2*) plus LINE-1. We found that methylation levels of the three genes were significantly lower in normal colonic mucosa than in the corresponding tumors (Supplementary Figure S2A–2C, S3A–2C), and that methylation did not correlate with location within the bowel (Figure 4A–4C, Supplementary Figure S4). In addition, methylation of the selected genes in normal-appearing colonic mucosa was not associated with the CIMP or *MLH1* methylation status in the corresponding tumor tissues (Supplementary Figure S5A–5C, S6A–6C).

By contrast, levels of LINE-1 methylation were lower in the lesions than the adjacent normal-appearing colonic mucosa (*P* = 0.02; Supplementary Figure S2D, S3D). Although levels of LINE-1 methylation in the normal colon did not correlate with location within the bowel or *MLH1* methylation (Figure 4D, Supplementary Figure S6D), colonic tissues adjacent to CIMP-positive lesions exhibited lower LINE-1 methylation levels than those adjacent to CIMP-negative lesions (*P* = 0.04, Supplementary Figure S5D). These results suggest changes in gene methylation occur in the normal colonic mucosa adjacent to tumors, but they do not strongly support the existence of an epigenetic field defect.

## DISCUSSION

In this study, we comprehensively analyzed *BRAF*-mutant colorectal lesions and observed an interesting relationship between epigenetic alterations within the lesions and their bowel subsite locations. The frequency of CIMP-positive lesions significantly differed between the proximal and distal colon when the sigmoid-descending colon junction was used as a borderline. Similarly, levels of DNA methylation in *RASSF2*, *SOX5, GALNT14* and *miR-34b/c*, four cancer-related genes, were strikingly higher in lesions located in the proximal colon (from the cecum to the descending colon) than in those in more distal subsites. This probably reflects the higher frequency of SSAs (including SSAs with dysplasia and TSAs with SSAs) in the region extending from the cecum to the descending colon. To prevent progression to CRC, it is recommended that all serrated lesions proximal to the sigmoid colon be resected during colonoscopy [11]. Our results support that recommendation.

Recent studies suggest TSAs represent a heterogeneous category containing lesions that developed through at least two different neoplastic progression pathways that are distinct from the SSA pathway [16, 17]. For instance, the majority of SSAs exhibit *BRAF* mutations, while TSAs exhibit *KRAS* or *BRAF* mutations [16–19]. It is still unclear whether *BRAF*-mutant TSAs and SSAs develop into CRCs via the same neoplastic pathway, though one recent study proposed these lesions follow the same molecular pathway (*BRAF* mutation pathway) [17]. From the viewpoint of molecular analysis, the combination of MAP kinase pathway activation and CIMP is potentially strong evidence supporting their continued inclusion [17]. In addition, the presence of non-dysplastic precursor lesions (HPs or SSAs) associated with TSAs, which is reportedly observed in approximately 20-50% of TSAs and is significantly associated with *BRAF* mutation, also supports this idea [17–20]. Consistent with earlier reports [17–19], 6 of 21 TSAs (28.5%) were associated with SSAs, and all of the lesions were in the proximal colon, while 13 of 15 TSAs without precursor lesions were located in the distal colon. Our analysis also revealed that the prevalence of CIMP in TSA+SSAs was equivalent to that in SSAs and SSA+CDs, which could contribute to the observed differences in epigenetic alterations in *BRAF*-mutant serrated lesions in different bowel subsites. It is still unclear whether TSAs arise from SSAs or whether TSA+SSAs are in the same category as SSA+CDs. A recent study showed that the SSA marker annexin A10 (ANXA10) is not expressed in TSAs or in adjacent SSAs, suggesting the precursor serrated polyps associated with TSA differ biologically from SSAs [20]. In this study, we also noted that the frequency of *CDKN2A* methylation is higher in SSA+CDs (4 of 9 cases, 44.4%) than in TSA+SSAs (1 of 6 cases, 16.7%), though the difference was not statistically significant. This suggests methylation profiles may differ between the two lesions, though more comprehensive analysis of DNA methylation will be required to draw a conclusion.

Cumulative evidence suggests *BRAF*-mutant SSAs are precursors of CRCs with MSI, while *BRAF*-mutant TSAs are reportedly precursors of microsatellite-stable (MSS) CRCs [16, 17]. In addition, recent studies showed that MSI/*BRAF*-mutant CRCs are associated with a favorable prognosis, while *BRAF*-mutant MSS CRCs were associated with a poor one [33–35]. These results suggest that the methylation status of cancer-related genes and the tumor locations are associated with the pathological findings, carcinogenic potentials and prognoses of *BRAF*-mutant lesions.

To assess the involvement of an epigenetic field defect in the development of *BRAF*-mutant lesions, we analyzed the DNA methylation status of genes in normal colonic mucosa adjacent to the lesions. An et al. reported that levels of *RASSF1A* and *SFRP1* methylation in normal-appearing mucosa from patients with distal CRCs or polyps (conventional adenomas) were significantly higher than in proximal CRC or polyp patients [29]. In the present study, however, we did not observe significant differences in gene methylation in normal-appearing colonic mucosa among specimens from individuals with lesions in different bowel subsites, which is in contrast to the findings of Kawakami et al [28]. In addition, Worthley et al. [30] reported that the mean CIMP Z-scores calculated from the pancolorectal percent of methylated reference (PMR) using a CIMP panel (*CACNA1G*, *IGF2*, *RUNX3*, *NEUROG1*, and *SOCS1*) in the background mucosa of advanced proximal serrated polyps (advanced PSPs: >1 cm in diameter or with advanced features, including SSA, TSA or mixed polyp) were higher than in colonic mucosa with non-advanced PSPs or without any polyps. We also compared the methylation levels in the background mucosa of advanced PSPs with the levels in background mucosa from other types of serrated polyps, but we found no significant differences (data not shown). This difference between our findings and those of Worthley et al. [30] likely reflects differences in the method of methylation analysis (*MethyLight* vs. pyrosequencing), lesions analyzed (all serrated lesions vs. *BRAF*-mutant lesions) and sampling locations (pancolorectal vs. adjacent mucosa).

The present study has several limitations, including a relatively small sample size, a limited number of lesions available for MSI analysis, the absence of normal background samples from subjects without lesions, and a limited number of genes assessed in the normal-appearing background samples. Nonetheless, we were able to make several important observations. First, methylation status in *BRAF*-mutant lesions is strongly associated with their location, histological findings and neoplastic pathways. Second, the methylation profiles in *BRAF*-mutant lesions in the proximal colon may greatly differ from those in the distal bowel when the sigmoid-descending colon junction is used as a demarcation point. Third, there were no significant differences in the methylation levels in background normal mucosa from different bowel subsites, which may indicate the absence of an epigenetic field defect for *BRAF*-mutant lesions. At present, it remains unclear whether SSAs and TSAs with *BRAF* mutations share a common origin. Further study to clarify the spectrum of genetic and epigenetic alterations in *BRAF*-mutant lesions, including SSAs with or without dysplasia, TSAs and TSA+SSAs, will likely provide new insight into the molecular mechanisms underlying different neoplastic pathways.

## MATERIALS AND METHODS

### Patients and tissue samples

Biopsy specimens from *BRAF*-mutant colorectal lesions (n = 106) and corresponding noncancerous colonic mucosa (n = 83) were obtained from 94 Japanese patients who underwent colonoscopic examination at Akita Red Cross Hospital (Table 1). Informed consent was obtained from all patients before collection of the specimens, and approval of this study was obtained from the Institutional Review Board of Akita Red Cross Hospital and Sapporo Medical University.

### Endoscopic analysis

High-resolution magnifying endoscopes (CF260AZI; Olympus, Tokyo, Japan) were used for all colonoscopic analyses. All lesions detected during colonoscopy were observed at high magnification using indigo carmine dye, after which biopsy specimens were collected from the lesions and adjacent normal-appearing colonic mucosa for genomic DNA extraction. The lesions were then treated by endoscopic mucosal resection, endoscopic submucosal dissection or surgical resection for histological analysis, as previously reported [13,14].

### Histological analysis

**Figure 5:**
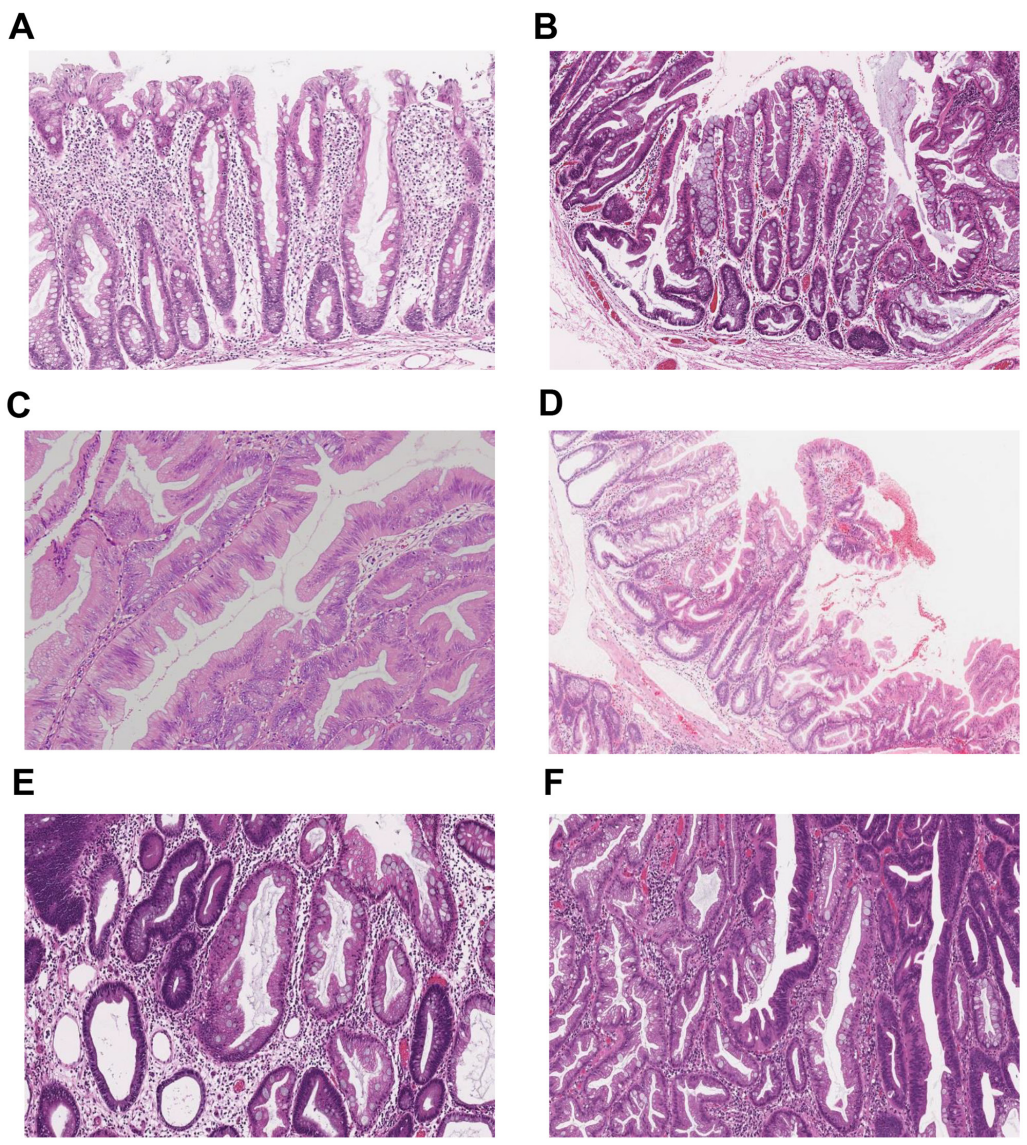
Representative histopathological images of the major serrated lesion subtypes **A.** Hyperplastic polyp (HP). **B.** Sessile serrated adenoma (SSA). **C.** Traditional serrated adenoma (TSA). **D.** TSA with SSA (TSA+SSA). **E.** SSA with cytological dysplasia (SSA+CD). **F.** SSA with high-grade dysplasia (SSA+HGD).

Histological findings for all specimens were evaluated by a pathologist (T.S.) who was blinded to the clinical and molecular information. Conventional adenoma was diagnosed using the standard criteria. Serrated lesions (HPs, SSAs and TSAs) were classified based on the criteria previously described by Torlakovic et al [9]. Mixed serrated lesions composed of SSA, TSA, CD and HGD were evaluated on the basis of each component and described as TSA+SSA, SSA+CD or SSA+HGD. The clinicopathological features of the lesions are summarized in Table 2, and salient histopathological features of the major polyp subtypes are shown in Figure 5. Tumor locations were classified as cecum, ascending colon, transverse colon, descending colon, sigmoid colon and rectum (rectosigmoid was classified into sigmoid colon).

### Mutation analysis

Genomic DNA was extracted using the standard phenol-chloroform procedure. Mutation in codon 600 of *BRAF* was examined by pyrosequencing using a *BRAF* pyro kit (Qiagen, Hiden, Germany) according to the manufacturer's instructions.

### DNA methylation analysis

DNA methylation was analyzed using bisulfite pyrosequencing as described previously [36]. Briefly, genomic DNA (1 μg) was modified with sodium bisulfite using an EpiTect Bisulfite kit (Qiagen). Pyrosequencing was then carried out using a PSQ 96MA system (Qiagen) with a Pyro Gold Reagent kit (Qiagen), and the results were analyzed using Pyro Q-CpG software (Qiagen). A cutoff value of 15% was used to define genes as methylation-positive. Tumors were defined as CIMP-positive when methylation was detected in three or more loci out of five classic CIMP markers (*MINT1*, *MINT2*, *MINT12*, *MINT31* and *MLH1*) and *CDKN2A* (*p16*). Methylation of *LRP1B*, *SOX5*, *GALNT14*, *RASSF2*, *IGFBP7*, *miR-34b/c*, *SFRP1*, *SFRP2* and long interspersed nucleotide elements (LINE-1) was also analyzed using bisulfite pyrosequencing. Primer sequences were as previously reported [14, 37].

### MSI analysis

MSI was assessed as described previously [38]. MSI status was determined by examining a panel of 5 microsatellite markers (BAT25, BAT26, D5S346, D2S123, D17S250) [39]. Lesions were defined as MSI-positive when two or more markers showed instability.

### Statistical analysis

Continuous data were analyzed using *t*-tests (for two groups) or ANOVA with a post hoc Tukey's HSD test (for more than two groups). Fisher's exact test and logistic regression were performed to assess the association between categorical variables. *P* < 0.05 was considered statistically significant. All statistical analyses were performed using SPSS 20 (IBM Corporation, Somers, NY) and GraphPad Prism 6 (GraphPad Software, La Jolla, CA).

## SUPPLEMENTARY FIGURES


