# Supplementary material for: Assessment of epigenetic alterations in early colorectal lesions containing BRAF mutations
Source: Oncotarget. 2016 Apr 27;7(23):35106–18. doi: 10.18632/oncotarget.9044 (PMC5085213; doi:10.18632/oncotarget.9044)
Supplement: Supplementary file 1 [file oncotarget-07-35106-s001.pdf]

SUPPLEMENTARY FIGURES

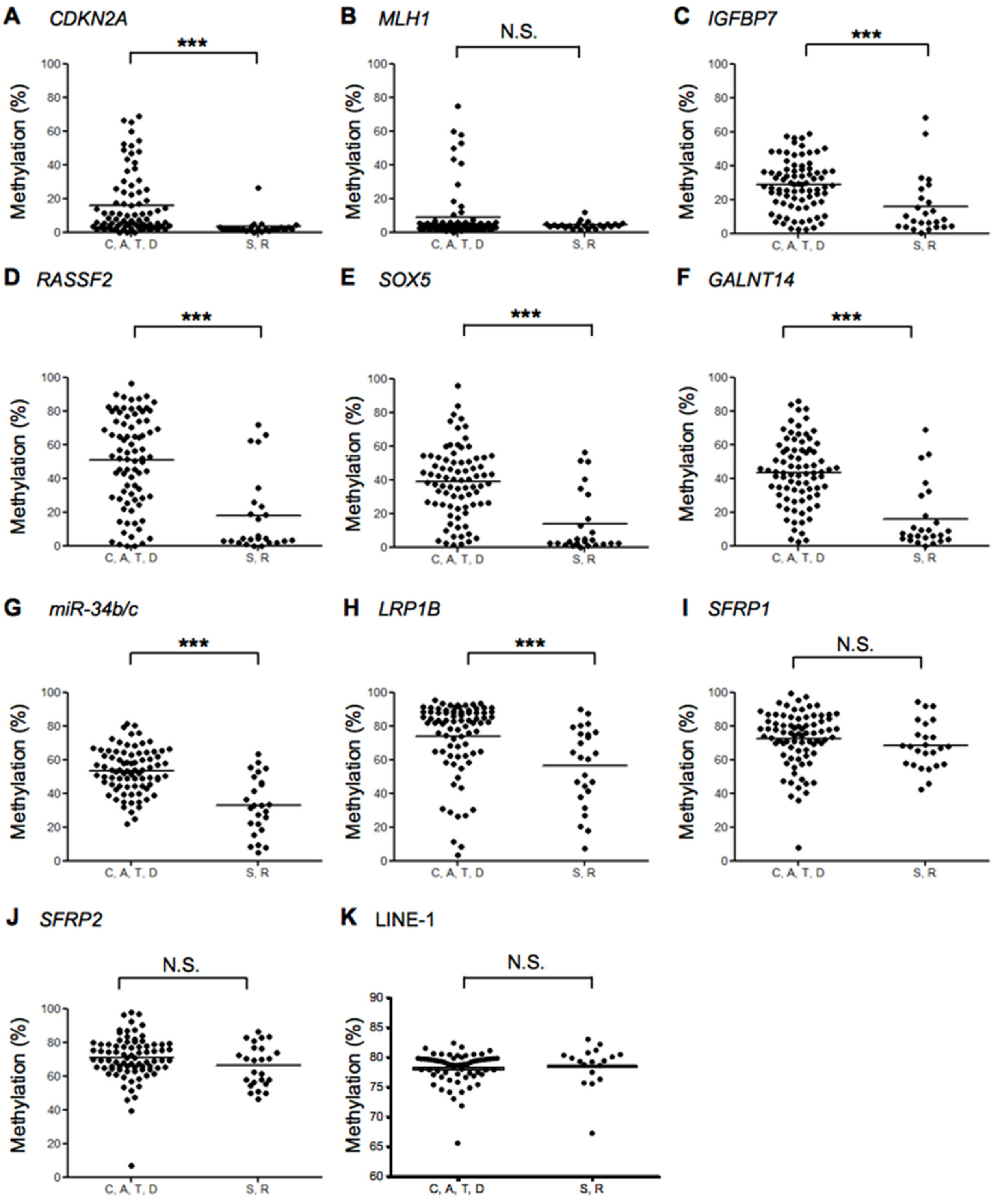

Supplementary Figure S1: Summaries of bisulfite pyrosequencing results for the indicated genes and LINE-1 in *BRAF*-mutant lesions in the proximal colon (cecum to descending colon) and distal colorectum (sigmoid colon and rectum). C, cecum; A, ascending colon; T, transverse colon; D, descending colon; S, sigmoid colon; R, rectum. ***P<0.001, N.S: not significant.

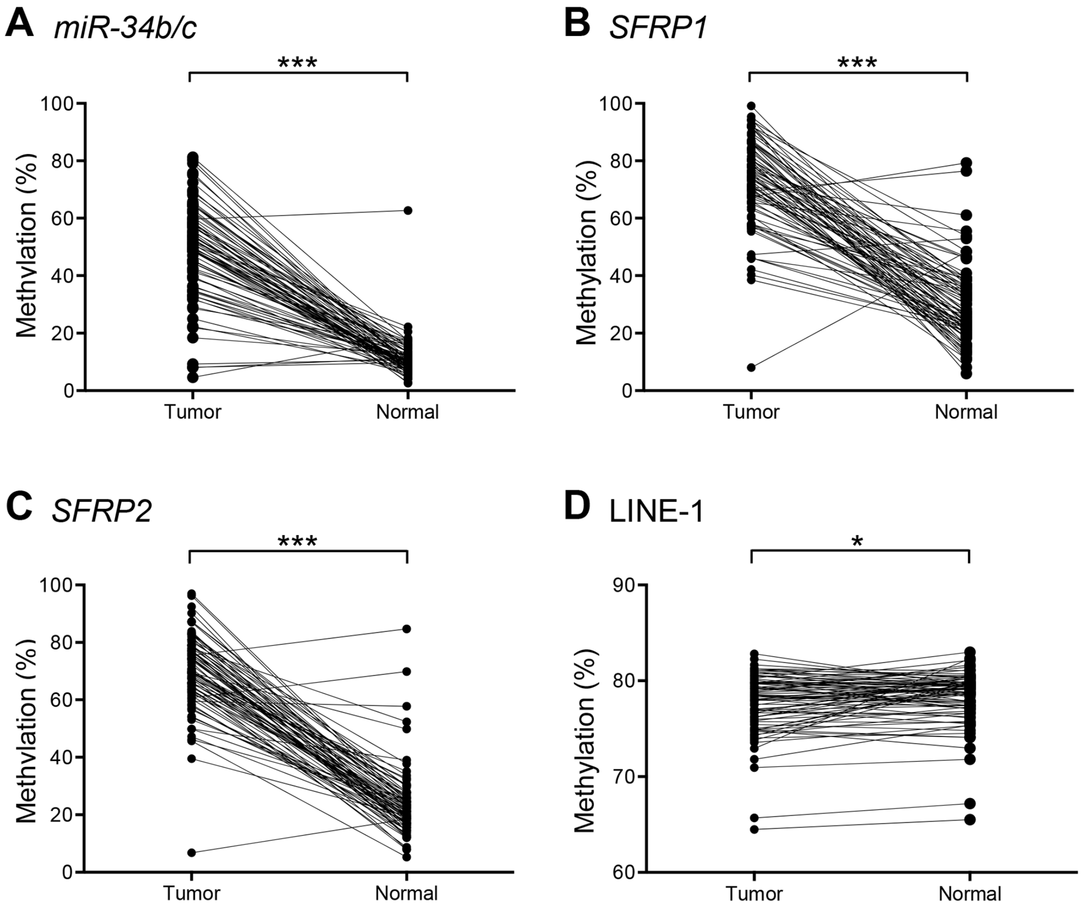

Supplementary Figure S2: Levels of methylation of the indicated genes and LINE-1 in *BRAF*-mutant lesions (Tumor) and matched normal-appearing mucosa (Normal) (n = 83). *$P < 0.05$, ***$P < 0.001$.

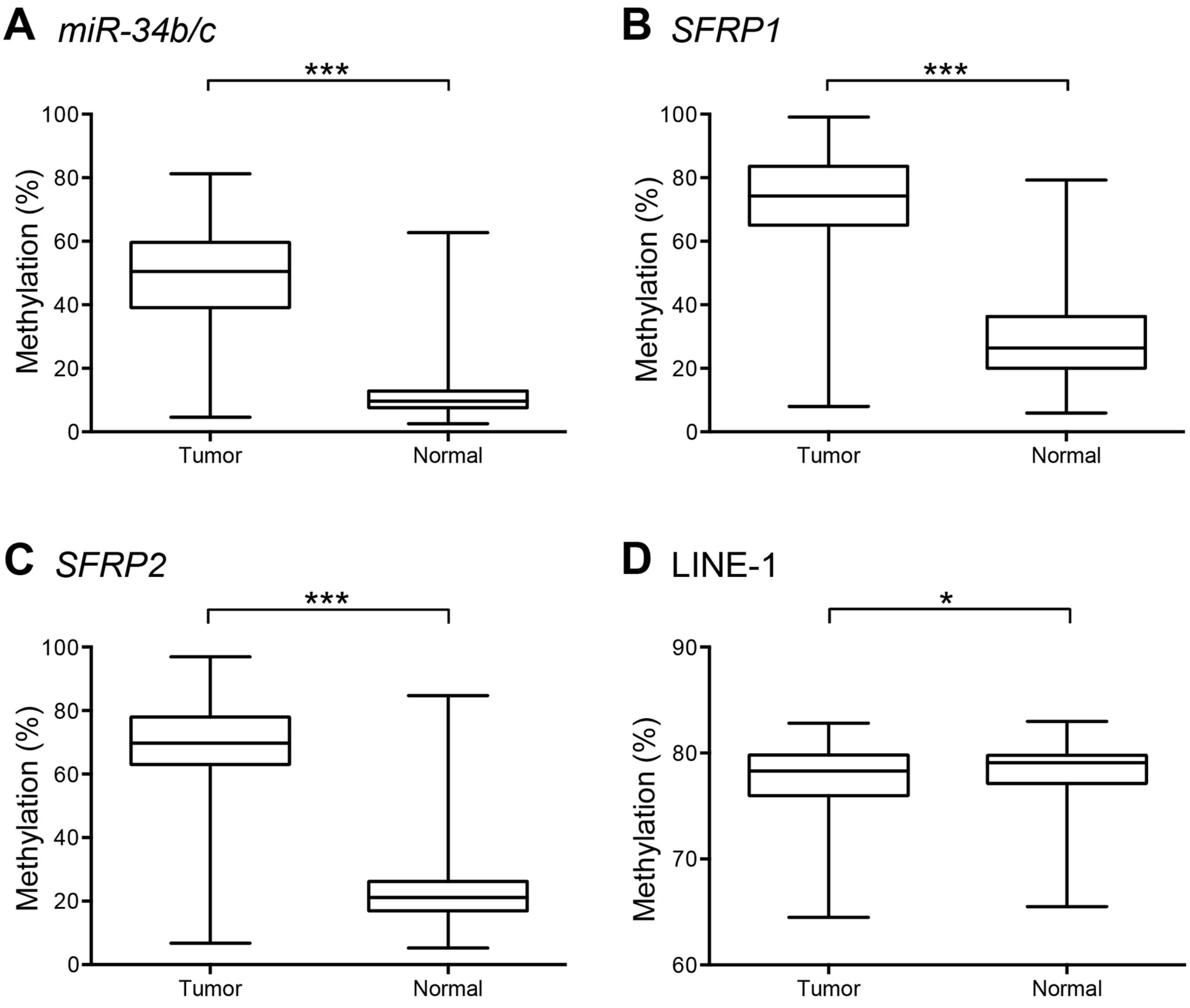

Supplementary Figure S3: Box-and-whisker plot showing the methylation levels of the indicated genes and LINE-1 in *BRAF*-mutant lesions (Tumor) and matched normal-appearing mucosa (Normal) (n = 83). *$P < 0.05$, ***$P < 0.001$.

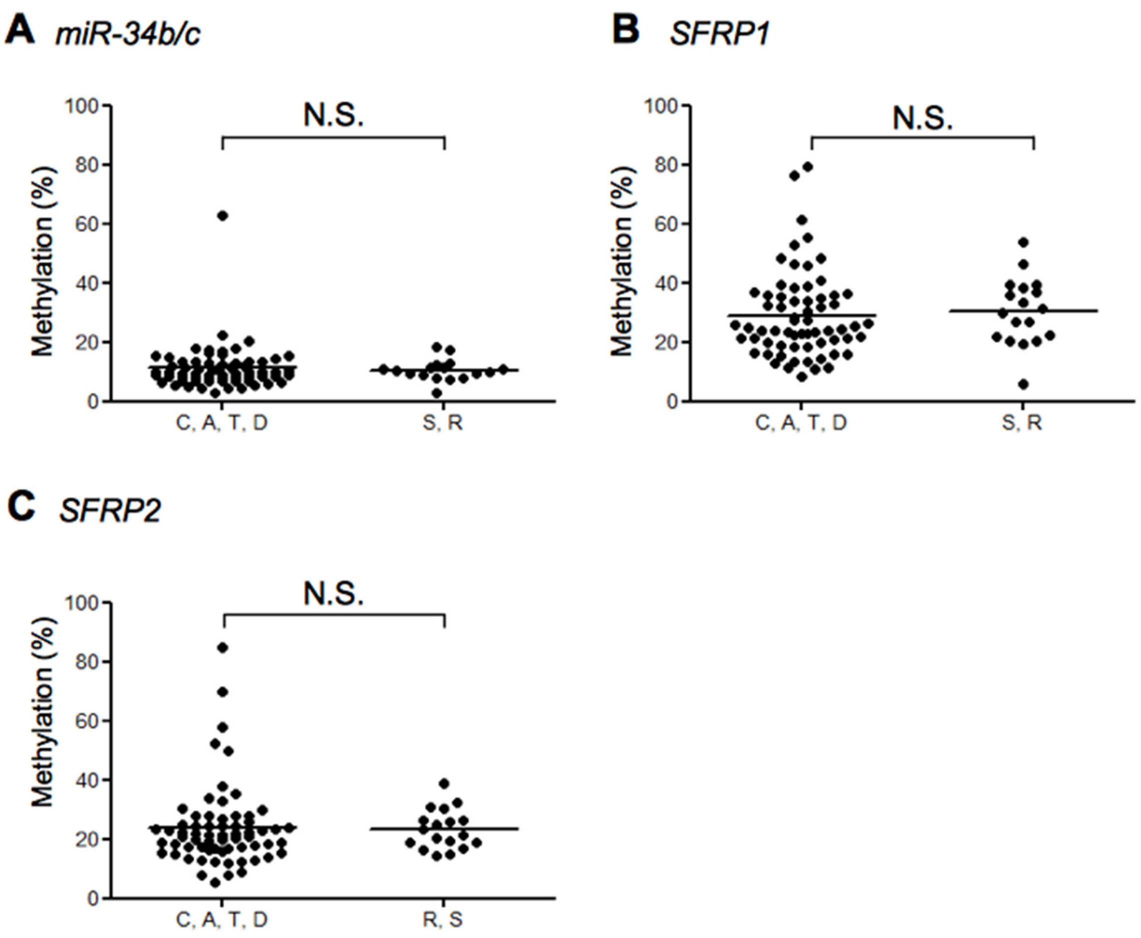

Supplementary Figure S4: Levels of methylation of the indicated genes in normal-appearing mucosa adjacent to *BRAF*-mutant lesions in the proximal colon (from cecum to descending colon) and distal colorectum (sigmoid colon and rectum). C, cecum; A, ascending colon; T, transverse colon; D, descending colon; S, sigmoid colon; R, rectum; N.S., not significant.

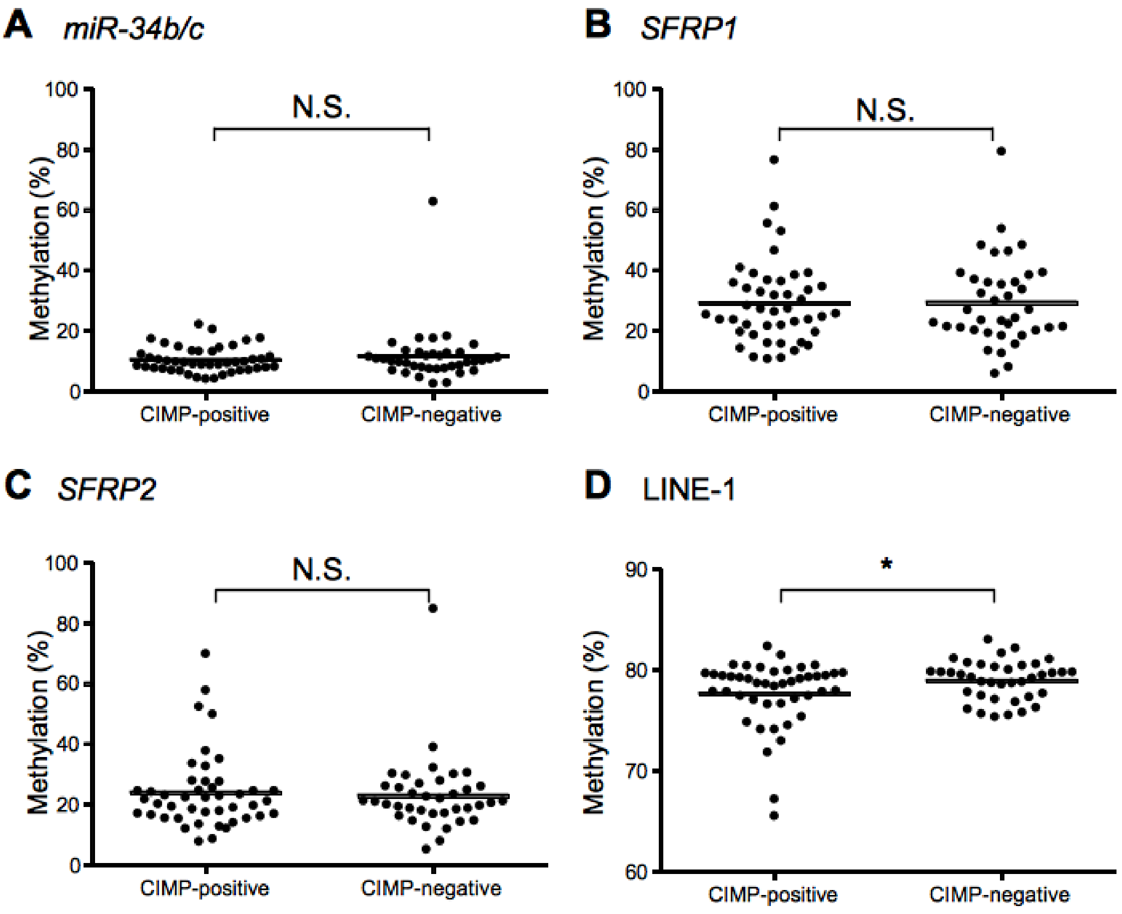

Supplementary Figure S5: Levels of methylation of the indicated genes and LINE-1 in normal-appearing mucosa adjacent to *BRAF*-mutant lesions with or without CIMP. *$P < 0.05$, N.S.: not significant.

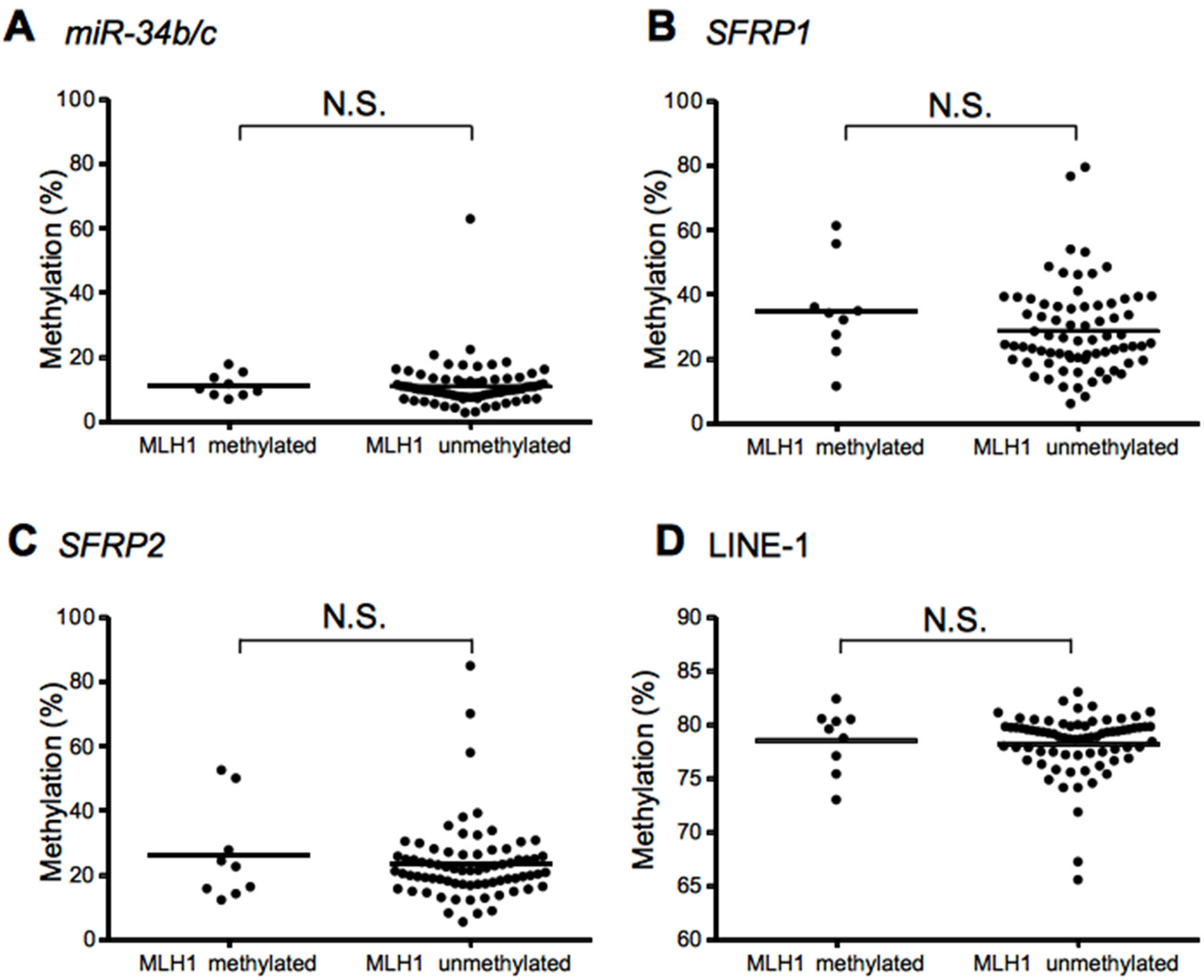

Supplementary Figure S6: Levels of methylation of the indicated genes and LINE-1 in normal-appearing mucosa adjacent to *BRAF*-mutant lesions with or without *MLH1* methylation. N.S.: not significant.
